# Integrated plasma proteomics and lung transcriptomics reveal novel biomarkers in idiopathic pulmonary fibrosis

**DOI:** 10.1186/s12931-021-01860-3

**Published:** 2021-10-24

**Authors:** Pitchumani Sivakumar, Ron Ammar, John Ryan Thompson, Yi Luo, Denis Streltsov, Mary Porteous, Carly McCoubrey, Edward Cantu, Michael F. Beers, Gabor Jarai, Jason D. Christie

**Affiliations:** 1 Translational Early Development, Bristol-Myers Squibb Research and Development, 3551 Lawrenceville Road, Princeton, NJ 08540 USA; 2 Informatics and Predictive Sciences, Bristol-Myers Squibb Research and Development, Princeton, NJ USA; 3 Translational Medicine, Bristol-Myers Squibb Research and Development, Princeton, NJ USA; 4 Fibrosis Discovery Biology, Bristol-Myers Squibb Research and Development, Princeton, NJ USA; 5 Pulmonary and Critical Care Medicine, University of Pennsylvania, Philadelphia, PA USA; 6 PENN Lung Biology Institute, University of Pennsylvania, Philadelphia, PA USA; 7 Department of Surgery, Division of Cardiovascular Surgery, University of Pennsylvania, Philadelphia, PA USA

**Keywords:** Idiopathic pulmonary fibrosis, Biomarkers, Chemokines, Mast cells, Extracellular matrix

## Abstract

**Background:**

Idiopathic pulmonary fibrosis (IPF) is a fatal lung disease with a significant unmet medical need. Development of transformational therapies for IPF is challenging in part to due to lack of robust predictive biomarkers of prognosis and treatment response. Importantly, circulating biomarkers of IPF are limited and none are in clinical use.

**Methods:**

We previously reported dysregulated pathways and new disease biomarkers in advanced IPF through RNA sequencing of lung tissues from a cohort of transplant-stage IPF patients (n = 36) in comparison to normal healthy donors (n = 19) and patients with acute lung injury (n = 11). Here we performed proteomic profiling of matching plasma samples from these cohorts through the Somascan-1300 SomaLogics platform.

**Results:**

Comparative analyses of lung transcriptomic and plasma proteomic signatures identified a set of 34 differentially expressed analytes (fold change (FC) ≥  ± 1.5, false discovery ratio (FDR) ≤ 0.1) in IPF samples compared to healthy controls. IPF samples showed strong enrichment of chemotaxis, tumor infiltration and mast cell migration pathways and downregulated extracellular matrix (ECM) degradation. Mucosal (CCL25 and CCL28) and Th2 (CCL17 and CCL22) chemokines were markedly upregulated in IPF and highly correlated within the subjects. The mast cell maturation chemokine, CXCL12, was also upregulated in IPF plasma (fold change 1.92, FDR 0.006) and significantly correlated (Pearson r = − 0.38, p = 0.022) to lung function (%predicted FVC), with a concomitant increase in the mast cell Tryptase, TPSB2. Markers of collagen III and VI degradation (C3M and C6M) were significantly downregulated (C3M p < 0.001 and C6M p < 0.0001 IPF vs control) and correlated, Pearson r = 0.77) in advanced IPF consistent with altered ECM homeostasis.

**Conclusions:**

Our study identifies a panel of tissue and circulating biomarkers with clinical utility in IPF that can be validated in future studies across larger cohorts.

**Supplementary Information:**

The online version contains supplementary material available at 10.1186/s12931-021-01860-3.

## Background

Idiopathic pulmonary fibrosis (IPF) is a progressive, chronic and fatal lung disease with a huge unmet medical need [1–3]. Despite the approval of two drugs that provide symptomatic relief, lung transplant remains the only option for long-term survival in IPF patients [4, 5]. Development of new drugs for IPF is extremely challenging due to complicated diagnosis, limited disease understanding, and a lack of biomarkers of disease progression and drug treatment [6]. This is further compounded by poor access to high quality and well annotated samples for translational biomarker studies. Transcriptomic and proteomic disease signatures generated from clinically relevant human samples including tissue and plasma, combined with robust “in silico” modeling can enable translational disease understanding, diagnosis and stratification of patients for effective drug treatments.

Past studies have profiled gene expression in lung tissues, peripheral blood and isolated cells through microarray and bulk/single cell RNA-sequencing analyses, identifying aberrant cell populations as well as molecular signatures of progressive IPF [7–12]. Recent IPF biomarker efforts have focused on identification of circulating biomarkers using plasma/serum or secreted biomarkers in matrices such as Broncho alveolar lavage, sputum and breath condensate obtained through minimally invasive procedures [13–15]. However, most studies have primarily used samples from progressive IPF patients where tissue biopsy is not in routine clinical practice. Given that IPF pathology is complex and involves interplay of tissue resident and infiltrating cells resulting in progressive and extensive tissue remodeling and scarring [16], it is possible that the peripheral biomarker signature may not accurately capture tissue level changes in advanced disease. For example, a crosslinked fibrotic extracellular matrix (ECM) could act as a barrier or trap preventing the detection of relevant disease biomarkers in circulation. Thus far, there have not been studies comprehensively examining tissue and plasma molecular signatures in unison. Using well annotated lung tissue samples from a cohort of transplant stage IPF patients in comparison to acute lung injury and healthy controls, we previously reported a transcriptomic fingerprint of advanced IPF enriched in pathways of T-cell activation, immune response and ECM remodeling [17]. These studies also identified novel gene associations to lung function as well as unique isoform regulation in IPF lung.

We hypothesized that a combined analysis of lung and plasma gene/protein signatures will identify robust biomarkers with potential clinical utility. Here, we have performed unbiased proteomic analyses of matching plasma from the advanced IPF cohort through the Somascan-1300 aptamer platform and compared the plasma proteome signature to the previously reported lung transcriptome signature. Our data reveal a striking enrichment of pathways involved in chemotaxis/Th2 chemokine and T-cell signaling, Wnt signaling, mast cell migration and activation, and extracellular matrix degradation in both tissue and plasma of advanced IPF. Notably, the Th2 chemokines CCL17 and CCL22 as well as mucosal chemokines CCL25 and CCL28 were robustly upregulated in IPF and correlated within subjects. The mast cell maturation chemokine CXCL12 was also increased in IPF together with a concomitant increase in the mast cell protease, TPSB2. Neoepitopes of collagen type III and VI degradation (C3M and C6M) were strongly downregulated and highly correlated in advanced IPF subjects. Our data provide a comprehensive signature of IPF tissue and plasma that could be potentially validated and utilized for clinical assessment of advanced IPF.

## Materials and methods

### Human subjects and sample acquisition

All human subject sample acquisitions and experiments were conducted with the appropriate approval from the Institutional Review Board (IRB 806468, IRB 813685). The clinical profile and demographics of IPF, ALI and control subjects used in this study have been previously described [17]. The IPF cohort consisted of 36 subjects with advanced IPF (mean % predicted forced vital capacity of 44) that underwent lung transplantation at the University of Pennsylvania. The ALI and control cohorts consisted of subjects whose donated lungs were deemed ineligible for lung transplantation. Explant samples were evaluated by an experienced thoracic pathologist who classified samples as ‘ALI’ based on the presence of diffuse alveolar damage or as ‘control’ if no abnormal pathology was present.

### RNA sequencing in lung tissues

Details on the RNA-sequencing method and analyses have been described previously in detail [17].

### SomaLogic proteome assay

Plasma samples were collected in Citrate EDTA tubes in operating room prior to explant, centrifuged at 4 ℃ at 3521 RPM for 10 min, and stored at − 80℃. Plasma samples were analyzed on the SOMAscan V2 multiplex proteomic assay (SomaLogic, Boulder CO)—an aptamer-based quantitative proteomic biomarker discovery platform which measures 1033 analytes [18, 19]. The assay covers a broad range of proteins associated with disease physiology and biological functions, including cytokines, kinases, growth factors, proteases and their inhibitors, receptors, hormones and structural proteins. Plasma samples were distributed randomly in 96-well microtiter plates and the assay operators were blinded to the identity of all samples. Assay results were reported in normalized relative fluorescence units (RFU).

### Plasma data analyses

Each sample in the study was normalized by aligning the mean to a common reference. Inter-plate and inter-run calibration were achieved by applying a multiplicative scaling coefficient to each SOMAmer. These scaling factors were calculated using the eight reference calibrators on each plate. Sample data were first normalized to remove hybridization variation within a run followed by median normalization across all samples to remove other assay biases within the run and finally calibrated to remove assay differences between runs. Log transformed RFU values were used to analyze differential expression of biomarkers across cohorts by using the Limma linear modeling framework for differential expression [20, 21]. Pathway analyses was performed with the Metacore Genego platform using differential protein signatures obtained with a cutoff of 1.5-fold change and 0.1 FDR (false discovery rate).

### Collagen neoepitope assays

Neoepitopes of collagen III and VI degradation (C3M and C6M) respectively were analyzed by previously described ELISA methods [22, 23].

### Statistical analyses

Statistical analyses of differential gene expression data using the R package has been described previously [17]. SomaLogic bulk proteome data were analyzed as described in the “plasma data analyses” section. Comparison of transcriptome and proteome data was achieved by generating analyte lists with a similar cutoff of Fold change ≥  ± 1.5 and FDR of ≤ 0.1. Correlation between analytes as well as analyte-FVC correlations were assessed using the Pearson correlation analyses. Differential expression of individual analytes (gene and protein) as well as collagen neoepitope data were analyzed by one-way ANOVA followed by Tukey’s post test with differences considered significant at P < 0.05.

## Results

### SomaLogic profiling of IPF plasma

**Fig. 1:**
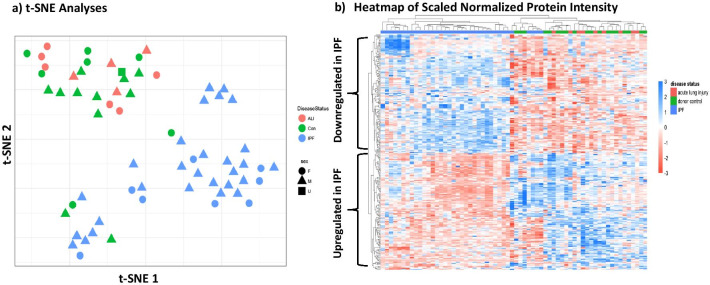
SomaLogics-based multiplex proteomic profiling of control, IPF and ALI plasma **a** Quality control analyses of Somalogics protein profiling data from IPF (n = 36), acute lung injury (n = 10) and control (n = 20) plasma, using the t-scholastic neighborhood enrichment (t-SNE method) showing clear separation of the IPF samples. **b** Proteomic fingerprint of advanced IPF plasma—differential protein expression between treatment groups was performed using the Limma package. Heatmap showing scaled intensity of individual proteins in rows and samples in columns. Note samples predominantly cluster based on disease status. *IPF* idiopathic pulmonary fibrosis, *ALI*  acute lung injury

Plasma samples from the IPF, ALI and healthy control cohorts were analyzed using the Somascan 1300-plex aptamer platform. Normalized log-transformed data were used to generate protein expression intensities. Expression data were visualized using t-scholastic neighborhood enrichment (t-SNE), that showed a robust separation of the IPF samples from the control and ALI samples (Fig. 1a). Further unbiased hierarchical clustering of the data revealed a strong clustering of the majority of IPF samples, thus revealing a proteomic fingerprint of advanced IPF (Fig. 1b). Notably, the ALI and control samples clustered together. These data suggested that the observed differences in protein intensities were primarily driven by the IPF disease state.

### Advanced IPF plasma proteome shows a strong signature of chemokine signaling, mast cell activation, Wnt signaling and extracellular matrix homeostasis

**Fig. 2:**
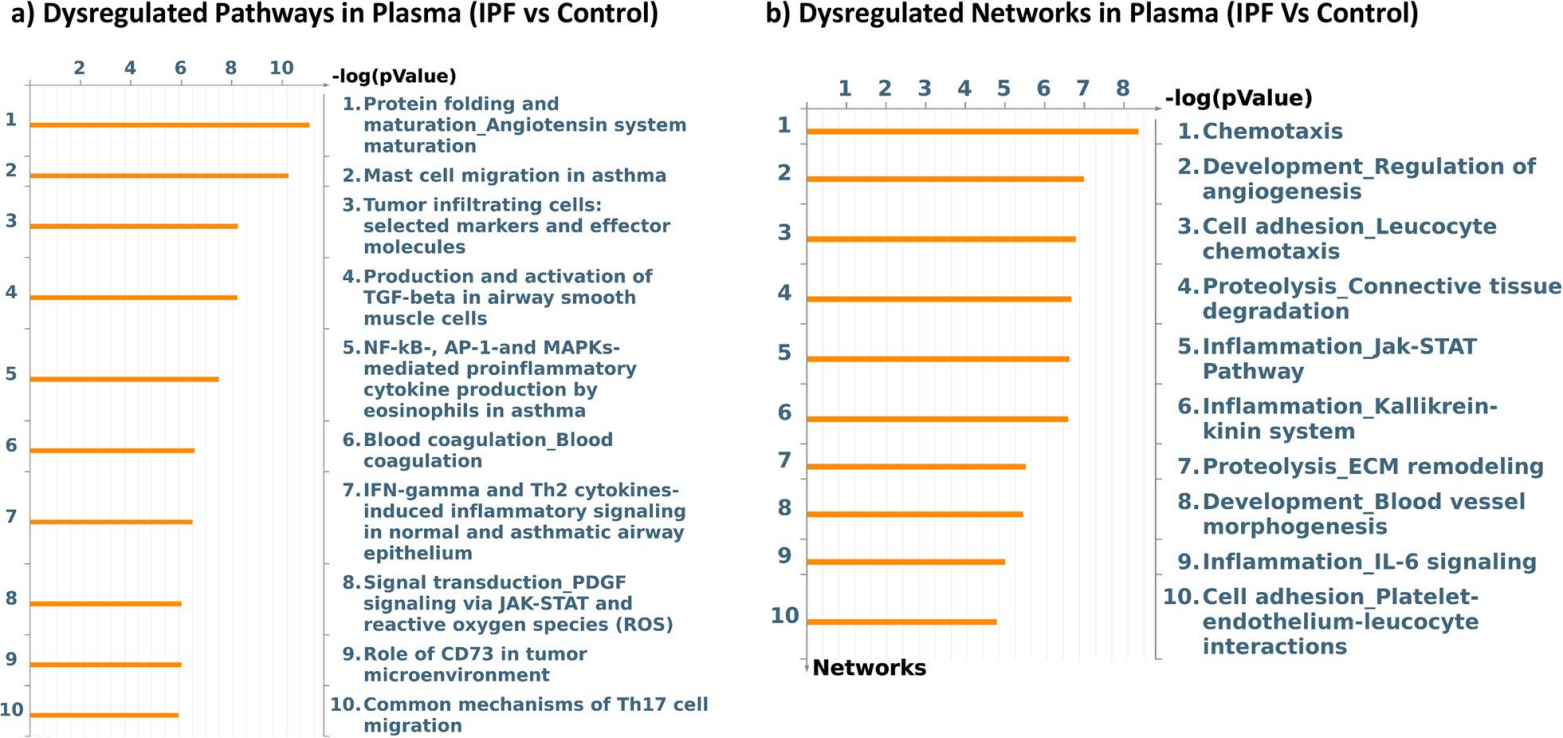
Differential pathway regulation in advanced IPF plasma. **a** Metacore analyses of pathway enrichment in IPF vs control plasma using a filter of positive and negative fold change ≥ 1.5 and adjusted P value of ≤ 0.1. **b** Top signaling networks enriched in IPF plasma compared to healthy controls

Using cutoffs of 1.5 and 0.1 for fold change and FDR respectively, we identified 236 differentially regulated proteins between IPF and control cohorts and 235 between IPF and ALI cohorts. Only two differentially regulated proteins were identified in the ALI vs control contrast. Therefore, subsequent analyses focused primarily on the differences between IPF and control cohorts. Tables 1 and 2 show the top 15 upregulated and downregulated proteins respectively in the IPF vs healthy contrasts (complete list of protein changes in IPF vs healthy contrast is provided in Additional file 1: Table S1). Metacore pathway analyses of differentially expressed proteins showed a striking modulation of chemotactic and immune pathways, mast cell migration and activity, as well as TGFβ signaling and ECM degradation and remodeling (Fig. 2a, b). As observed previously in our lung transcriptome signature, pathways involved in T-cell activation were distinctly upregulated in IPF plasma (Fig. 2a). Particularly interesting was the marked increase in a variety of chemokines involved in T-cell and other immune cell signaling. The eosinophilic chemokine CCL11, mucosal chemokines, CCL25 and CCL28, and the Th2 chemokines CCL17 and CCL22 were strongly upregulated in IPF plasma. The mast cell and lymphocyte chemoattractant SDF1/CXCL12, mast-cell derived chemokine CCL21, and the mast cell tryptase, TPSB2 were all markedly increased in IPF plasma. The Wnt signaling enhancers SPON1 and RSPO3 were also significantly increased together with a concomitant increase in the Wnt receptor, Frizzled B. Pathway analyses also revealed a marked regulation of ECM remodeling networks with a reduction in ECM remodeling proteases such as TIMP1 and SERPINs and an increase in profibrotic matrix molecules such as SPARC and Vitronectin (Fig. 2b). These changes were consistent with an advanced fibrotic disease state in our IPF cohort, where ECM synthesis is expected to significantly exceed degradation.

**Table 1:** Top proteins upregulated in IPF plasma compared to healthy controls

Gene	Description	Log fold change	FDR
CCL28	C-C motif chemokine 28	2.259556173	0.000231
CCL25	C-C motif chemokine 25	2.073155182	0.000257
TLR4	Toll-like receptor 4	1.856929692	0.00108
CCL11	Eotaxin	1.799567355	8.20E−05
RSPO3	R-spondin-3	1.729221937	0.004217
UNC5D	Netrin receptor UNC5D	1.689658186	0.000147
CCL17	C-C motif chemokine 17	1.621455261	0.000257
TNFSF12	Tumor necrosis factor ligand superfamily member 12	1.578591635	2.52E−06
FRZB	Secreted frizzled-related protein 3	1.506003793	0.001032
CCL21	C-C motif chemokine 21	1.40240372	0.000945
CXCL9	C-X-C motif chemokine 9	1.377906373	0.004835
GFRA1	GDNF family receptor alpha-1	1.307361522	0.048836
MDK	Midkine	1.272550426	0.035844
PRL	Prolactin	1.271289238	8.20E−05
CCL4L1	C-C motif chemokine 4-like	1.25625883	0.054414
MAP2K4	Dual specificity mitogen-activated protein kinase 4	1.218081304	0.006491
PDGFB	Platelet-derived growth factor subunit B	1.17880283	0.026191
CCL5	C-C motif chemokine 5	1.145110861	0.010668
AKT2	RAC-beta serine/threonine-protein kinase	1.126278423	0.000147
ICAM5	Intercellular adhesion molecule 5	1.098941547	0.000257
CA6	Carbonic anhydrase 6	1.094703466	0.061433
SPON1	Spondin-1	1.061155009	0.025752
CXCL11	C-X-C motif chemokine 11	1.059045713	0.056945
OLR1	Oxidized low-density lipoprotein receptor 1	1.05710928	0.077666
CTSV	Cathepsin L2	1.026447098	0.000346

**Table 2:** Top proteins downregulated in IPF plasma compared to healthy controls

Gene	Description	Log fold change	FDR
GFAP	Glial fibrillary acidic protein	− 3.660959317	8.20E−05
IL1RL1	Interleukin-1 receptor-like 1	− 2.634896607	0.000167
BMPR1A	Bone morphogenetic protein receptor type-1A	− 2.467593733	0.000692
SAA1	Serum amyloid A-1 protein	− 2.366367299	0.014601
BCAN	Brevican core protein	− 2.239288875	0.001523
HIST3H2A	Histone H2A type 3	− 2.206995388	0.00026
H2AFZ	Histone H2A.z	− 2.040764986	0.000545
NPPB	N-terminal pro-BNP	− 1.801131878	0.022043
PRSS2	Trypsin-2	− 1.678546737	0.033673
IGHE	Immunoglobulin E	− 1.639149251	0.026242
CHI3L1	Chitinase-3-like protein 1	− 1.550724228	0.035989
REG1A	Lithostathine-1-alpha	− 1.524188965	0.001408
PLA2G2A	Phospholipase A2; membrane associated	− 1.519910158	0.037611
IGFBP2	Insulin-like growth factor-binding protein 2	− 1.517034902	0.000329
EPO	Erythropoietin	− 1.307917165	0.02137
CKM	Creatine kinase M-type	− 1.262079547	0.040541
CD177	CD177 antigen	− 1.261375377	0.013795
CTSD	Cathepsin D	− 1.247292872	0.000277
TIMP1	Metalloproteinase inhibitor 1	− 1.213901397	0.000257
ANGPT2	Angiopoietin-2	− 1.208573769	8.20E−05
ACY1	Aminoacylase-1	− 1.203247133	0.014948
THBS2	Thrombospondin-2	− 1.193185641	0.06772
RETN	Resistin	− 1.166268536	0.000407
LDHB	L-lactate dehydrogenase B chain	− 1.123231706	0.009274
CA3	Carbonic anhydrase 3	− 1.09182128	0.09789

### Correlation of pathway markers within the IPF cohort

**Fig. 3:**
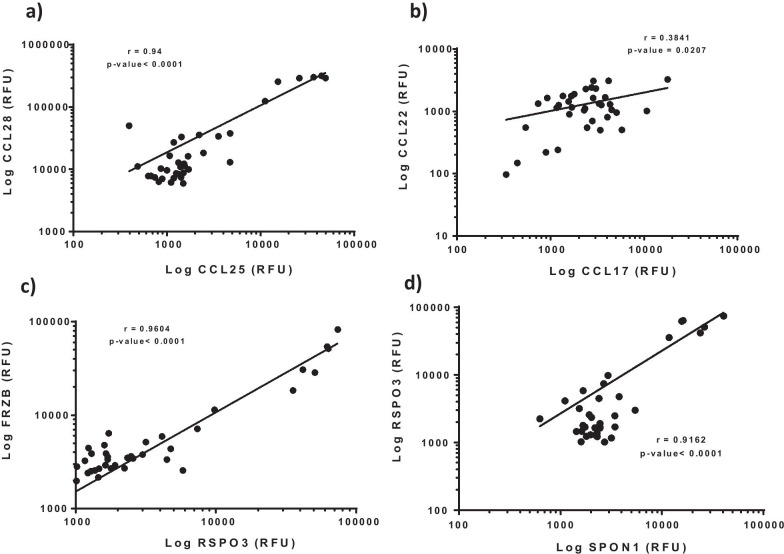
Correlation between components of multiple dysregulated pathways within individual subjects in IPF plasma (n = 36). Significant positive association between expression of the mucosal chemokines CCL25 and CCL28 (**a**), the T-helper 2 cytokines CCL17 and CCL22 (**b**), the Wnt signaling components FRZB, RSPO3 and SPON-1 (**c**, **d**). All correlations refer to Pearson correlation analyses

Intrigued by the strong enrichment of chemotactic and profibrotic signaling pathways in the IPF plasma, we sought to analyze the relationship between multiple components of the regulated pathways within the IPF cohort. These analyses showed that the expression of the mucosal chemokines CCL25 and CCL28 as well as that of the Th2 cytokines CCL17 and CCL222 were significantly correlated within the IPF subjects (Fig. 3a, b). Likewise, Wnt pathway molecules RSPO3, FRZB, and SPON1 were also strongly and significantly correlated within the IPF cohort (Fig. 3c, d). These data further support the findings that the indicated pathways were strongly dysregulated within the IPF cohort.

### Comparison of lung transcriptome and plasma proteome in IPF

**Fig. 4:**
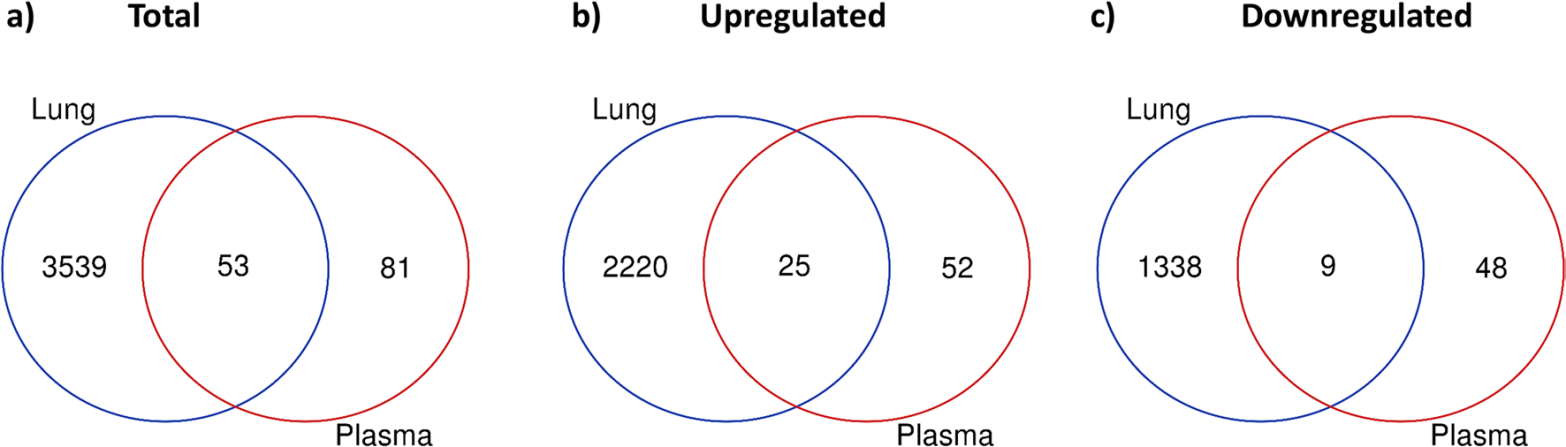
Comparison of lung transcriptome (RNA-seq) and plasma proteome (Somalogics) in advanced IPF. Differential contrasts between IPF and healthy samples were computed using cutoffs of FC ≥ 1.5 and adjusted P value of ≤ 0.1 for both the RNA-seq and Somalogics datasets. Venn diagram represents the intersection of the total signature (**a**), as well as gene/protein sets commonly upregulated (**b**) and downregulated (**c**) in lung and plasma

**Fig. 5:**
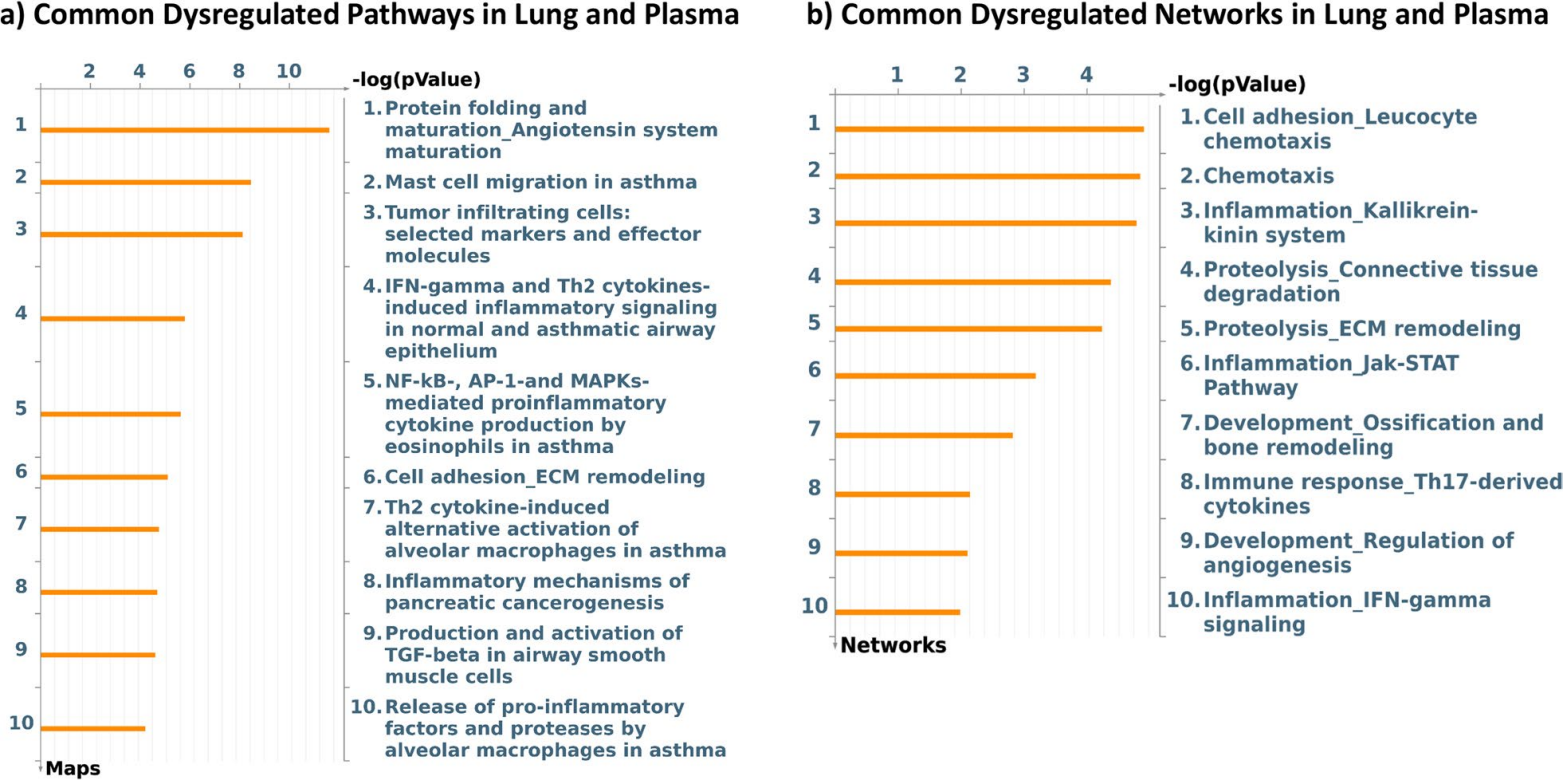
Commonly dysregulated pathways and networks in advanced IPF lung and plasma. Differential contrasts were generated using a cutoff of positive and negative fold change ≥ 1.5 and adjusted P value of ≤ 0.1. Resultant gene lists were analyzed by Metacore pathway analyses to identify pathways (a) and networks (b) commonly dysregulated in both lung and plasma

**Fig. 6:**
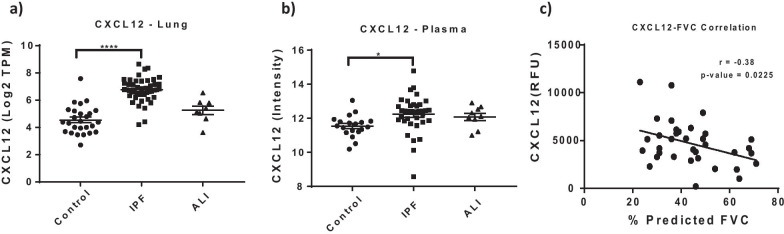
Identification of CXCL12 as a novel biomarker of advanced IPF **a** Increased gene expression of CXCL12 in IPF lung compared to healthy controls (**** p < 0.0001 vs control one way ANOVA and Tukey’s post test). **b** Increased protein expression of CXCL12 in IPF plasma compared to healthy controls (* p < 0.05 vs IPF, one way ANOVA and Tukey’s post test). **c** Plasma expression of CXCL12 is significantly correlated to lung function (% predicted FVC) in advanced IPF (Pearson’s correlation analyses)

Our IPF cohort offered the unique opportunity to perform an integrated analyses and comparison of the lung transcriptome signature and the plasma proteomic signature in unison. These analyses provided some insightful data into pathways and markers commonly and divergently dysregulated in tissue and peripheral blood. Using similar fold change and FDR cut-offs (FC ≥ 1.5, FDR ≤ 0.1), we identified 53 genes commonly regulated in lung and plasma, with 34 of them moving in the same direction (Fig. 4). The intersection of these signatures revealed a striking modulation of pathways involved in chemotaxis, T-cell activation, mast cell migration and activation, TGF beta signaling, Wnt activation and ECM homeostasis (Fig. 5). Tables 3 and 4 show that list of genes commonly up or down regulated in IPF lung and plasma. Most of these proteins were either chemokines or chemotactic factors or proteins involved in fibrotic signaling and ECM remodeling. Notably, the receptors for several of the upregulated chemokines in plasma were concomitantly increased in the lung transcriptome (previously published Additional file 1: Table S1 from [17]). These include CCR4 (receptor for CCL17 and 22), CCR7 (receptor for CCL21), CCR5 (receptor for CCL5), CCR10 (receptor for CCL28) and CXCR4 (receptor for CXCL12). The matricellular protein, SPARC (FC 1.58, FDR 0.005), and vitronectin (FC 1.52, FDR 0.074) were both increased in IPF plasma and lung indicative of an active profibrotic state. A particularly interesting finding in this study was the marked dysregulation of mast cell activators and mediators such as CCL21, CXCL12, CCL5 and Tryptase beta 2, that are known to promote a profibrotic response. Figure 6 shows that CXCL12 expression was increased in both lung (FC 6.68, FDR < 0.00001, Fig. 6a) and plasma (FC 1.92, FDR 0.006, Fig. 6b) and the plasma expression was significantly correlated with % predicted FVC (r = − 0.38, p = 0.022, Fig. 6c).

**Table 3:** Top proteins commonly upregulated in both lung tissue and plasma of IPF patients compared to healthy controls

Gene	Log fold change (lung)	FDR (Lung)	Log fold change (Plasma)	FDR (Plasma)
CCL11	4.484	0.00000000	1.799567355	8.20E−05
CCL22	3.569	0.00000067	0.87049819	0.035988956
CXCL12	2.740	0.00000000	0.943963155	0.006010717
TPSB2	2.449	0.00000002	0.724432917	0.079124595
MDK	2.396	0.00000000	1.272550426	0.035843766
KLK7	2.275	0.00088573	0.842680094	0.02034195
CCL21	2.019	0.00000000	1.40240372	0.000945317
SERPINA5	1.953	0.00010138	0.859861017	0.049974592
FRZB	1.939	0.00000000	1.506003793	0.00103215
GFRA1	1.820	0.00000000	1.307361522	0.048836237
VTN	1.736	0.00000901	0.609196466	0.074839355
CCL5	1.727	0.00000003	1.145110861	0.010668158
CCL3L1	1.654	0.00101204	0.60079124	0.027908725
CXCL9	1.591	0.00458221	1.377906373	0.00483477
RSPO3	1.404	0.00000191	1.729221937	0.0042172
SORCS2	1.247	0.00000009	0.890594785	0.049606009
CCL17	1.163	0.01754183	1.621455261	0.000257494
CTSV	1.129	0.00015761	1.026447098	0.000346371
GZMA	0.992	0.00104687	0.881790954	0.00103215
PDE5A	0.958	0.00000004	0.674159636	0.057080731
A2M	0.839	0.00000012	0.63442842	0.000928021
CTSF	0.737	0.00002812	0.647455699	0.004900564
CHL1	0.717	0.00338922	0.868595763	0.003669314
MSTN	0.677	0.00404260	0.716627957	0.034902232
SPARC	0.631	0.00001012	0.668856674	0.005760412

**Table 4:** Top proteins commonly downregulated in both lung tissue and plasma of IPF patients compared to healthy controls

Gene	Log fold change (lung)	FDR (Lung)	Log fold change (Plasma)	FDR (Plasma)
IL1RL1	− 3.544	0.000000000105	− 2.634896607	0.0001669
IL1R2	− 3.001	0.000000002738	− 1.033449865	0.031564344
S100A12	− 2.829	0.000000000327	− 1.073609496	0.044386712
IL18R1	− 2.173	0.000000000000	− 0.722860241	0.074670624
CD177	− 1.264	0.016722589668	− 1.261375377	0.013794617
RETN	− 1.205	0.000632239048	− 1.166268536	0.00040745
TNNT2	− 1.012	0.000535234220	− 0.941703387	0.035988956
FSTL3	− 0.717	0.000055605556	− 0.89064698	0.024740285
HIST2H2BE	0.117	0.633132897915	− 0.630306445	0.080621623

### Downregulated ECM degradation in IPF

**Fig. 7:**
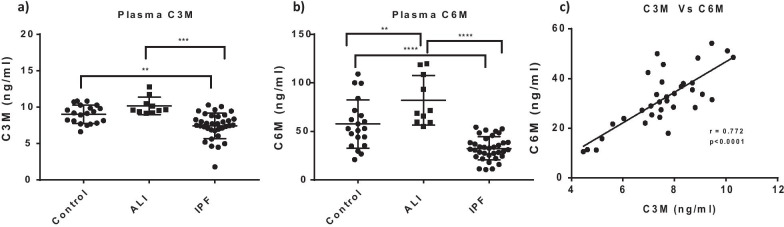
Decreased ECM degradation in advanced IPF **a** Decreased expression of C3M, collagen III neoepitope and **b** decreased expression of C6M, collagen VI neoepitope in IPF plasma compared to healthy controls. **c** Significantly positive correlation between plasma C3M and C6M within the IPF cohort. (**a** and **b**—** p < 0.01, *** p < 0.001, **** p < 0.0001, one way Anova and Tukey’s post test. **c**—Pearson correlation analyses)

**Fig. 8:**
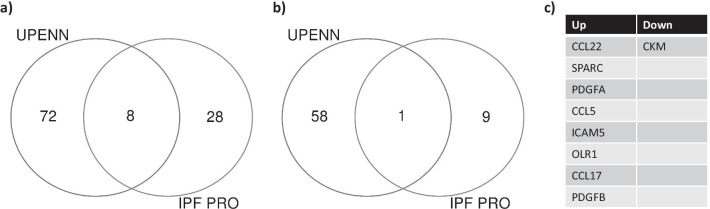
Comparison of advanced IPF proteome with Somalogics signature from IPF-PRO registry Venn diagram showing common and divergent proteins identified as upregulated (**a**) and downregulated (**b**) in both data sets **c**. List of biomarkers commonly altered in the UPenn and IPF-PRO registry datasets

Since pathway analyses of IPF plasma revealed a strong dysregulation of proteins involved in ECM homeostasis and remodeling, we measured C3M and C6M, neoepitopes of Collagen III and Collagen VI degradation respectively. Interestingly, we found a marked decrease in the levels of both C3M and C6M in IPF compared to healthy controls (Fig. 7a, b). Additionally, we found a high degree of correlation between the expression of both markers within the IPF cohort. We finally compared our plasma proteome signature to a recently published Somalogics plasma signature from a cohort of IPF patients from the IPF PRO registry [24] and found SPARC, CCL5, CCL17 and CCL22, OLR1 and PDGF-a/B as commonly regulated in similar directions in both IPF datasets (Fig. 8a-c).

## Discussion

We describe here for the first time a simultaneous comprehensive analyses of plasma proteome and lung transcriptome of a unique cohort of advanced IPF patients in comparison to that of normal healthy donors. Our studies suggest a strong dysregulation of T-cell activation, chemokine signaling, mast cell maturation, Wnt signaling and ECM homeostasis pathways in lung tissue as well as peripheral blood of these patients and identify new biomarkers that could have clinical utility. Although numerous profiling studies in the past have identified dysregulated genes, proteins and pathways in early and progressive IPF, there are currently no diagnostic or prognostic biomarkers in clinical practice [6, 24–26]. This particular cohort of transplant-stage IPF patients provided an opportunity to compare and correlate tissue and plasma signatures in unison.

Unbiased hierarchical clustering of protein expression across the cohorts shows that the IPF samples were clearly separated from control and ALI samples. Consistent with this separation, we did not find major differences between the ALI and control cohorts, although our previously published RNA-seq data identified significant differences between the cohorts at the gene expression level. In the ALI samples, the degree of diffuse alveolar damage varied with the majority having extensive areas of pathology, while some had more focal areas. This degree of sample heterogeneity is expected and may have potentially contributed to the similarities seen between the ALI and donor sample groups in our study.

Plasma data from the IPF cohort not only confirmed and extended our previous lung transcriptome findings in the same cohort [17], but also provided potential insights into the key pathways and markers that could be involved in IPF disease progression. We observed a strikingly enhanced chemokine signaling signature in our IPF cohort, spanning a diverse group of chemokine-receptor pairs that contribute to both inflammation and tissue remodeling. CCL17/TARC and CCL22 are thymic chemokines previously shown to be upregulated in IPF BAL fluid and correlated to CCR4 expressing alveolar macrophages [27]. CCL28 is a classic mucosal chemokine known to signal through the CCR10 receptor, and CCR10 + epithelial cells are known to drive IPF progression [28]. CCL21 signaling through CCR7 expressed on activated IPF fibroblasts enhances fibrogenesis and neutralization of this pathway attenuates fibrosis [29, 30]. Although the role of eosinophils in IPF is poorly understood, it is known that eosinophils promote fibrotic airway remodeling and collagen deposition in allergic inflammation [31]. Emerging evidence also indicates that pathogenic memory Th-2 cells can activate eosinophils to produce profibrotic factors such as osteopontin [32]. The identification of multiple chemokine subtypes in our study could suggest that interplay of chemokine signaling through the mucosal, epithelial and Th-2 axis could together potentiate several pathogenic mechanisms in IPF including macrophage activation, T-lymphocyte homing, epithelial plasticity, and eosinophil influx. While shifting paradigms over the years have suggested dissociation of early inflammation from advanced fibrosis in IPF, our current findings suggest that inflammatory mechanisms remain active in advanced disease. Recently, artificial intelligence based approaches have also identified mononuclear inflammation, alveolar macrophages and fibroblast foci as potential prognostic biomarkers of IPF [33]. In addition to the increased expression of chemokines, we also show positive correlation between multiple chemokines in several pathways within the IPF cohort, further emphasizing the potential role of these pathways in disease progression. Intriguingly, the Th2 and eosinophilic signature in our IPF cohort was closely similar to the hallmarks of allergic inflammation as seen in asthmatic airways [34], further corroborated by a dominance of asthma-related mechanisms in our pathway analyses.

Another key finding from our study was the upregulation of the mast cell chemokines, CCL5, CCL21 and CXCL12, and the mast cell protease tryptase-B2. Prior studies have shown increased infiltrating mast cell numbers and tryptase activity in human IPF [35, 36], and therapeutic targeting of CXCL12/CXCR4 signaling attenuated bleomycin induced lung fibrosis in mice [37, 38]. Additionally, blood levels of CXCL12 as well as CXCR4 + cells within the honeycomb cysts and distal epithelium in tissue are increased in IPF [39]. Recent data also suggest that the antifibrotic drug, Nintedanib, could work through inhibition of mast cell survival and activity [40]. Notably, in our study we show that CXCL12 is not only increased in both lung and plasma but also correlated with % predicted FVC, suggesting that CXCL12 could be a tractable disease biomarker of advanced IPF. Mast cells have been long recognized to promote allergic inflammation, fibroblast activation and subepithelial fibrosis in asthma [41, 42]. Our collective findings in lung and plasma could imply that mast cell activation and degranulation could provide profibrotic mediators, growth factors and proteases that can potentially activate fibroblasts and impact ECM remodeling in advanced IPF.

Aberrant reactivation of developmental pathways including that of Wnt signaling is known to play a role in the pathogenesis of IPF [43]. In our study, we found a concomitant increase in several components of Wnt signaling including the Wnt activators R-spondin 3 (RSPO3) and SPON1 and the Wnt receptor, FRZB in both lung tissue and plasma. Additionally, the expression of these proteins was significantly correlated within the individual subjects in the IPF cohort. The increase in RSPO3 was particularly interesting in the light of a recent report that therapeutic targeting of RSPO3 attenuates fibrosis in multiple organs such as lung, liver and skin [44].

Consistent with an advanced fibrotic state, we found ECM remodeling and proteolysis pathways strongly dysregulated in the IPF cohort. The matricellular protein, SPARC, plays a key role in promoting collagen assembly into the ECM, and implicated as a biomarker in previous studies [24, 45]. SPARC gene and protein expression were strongly upregulated in our IPF cohort. Enrichment of ECM proteolysis pathways in IPF plasma is consistent with our previous findings on downregulated ECM degradation in the tissue. A surprising finding in our study was a strong downregulation of neoepitopes of collagen III and VI degradation, C3M and C6M respectively. Baseline levels of C3M and C6M are known to be predictive of progressive fibrosis and are elevated in newly diagnosed IPF patients [46, 47]. However, our IPF cohort represents a significantly advanced IPF population in which it is possible that extensive ECM turnover during the course of the disease would have resulted in a net increase in synthesis and reduction in degradation leading to a potential decrease in these markers. It is also possible that advanced IPF lung tissue is highly crosslinked, and less susceptible to degradation and turnover or that the matrix could act as a barrier to the release and subsequent identification of these markers in circulation. Supporting this notion, our lung RNA-seq data clearly shows marked upregulation of pro-fibrotic and synthetic ECM proteins and a downregulation of ECM degradation pathways. Furthermore, C3M and C6M were significantly correlated among the IPF subjects suggesting an overall decrease in ECM degradation in advanced IPF. Our data also could imply that the dynamics of ECM turnover could be different through the progression of IPF resulting in potential temporal differences in the levels of neoepitopes of ECM synthesis and degradation.

Although RNA-seq and SomaLogics represent distinctly different platforms that limit robust comparison, our analyses clearly confirmed many analytes dysregulated at gene level to be differentially regulated in plasma as well. It is possible that many other markers of interest may be missed due to the targeted 1300-plex analyses. Future studies with the currently available expanded Somascan platform (~ 7000 analytes) could help further identify and validate additional biomarkers of disease. Interestingly, new emerging data indicates that the use of a multi-omic approach such as ours could be valuable in identifying molecular disease signatures and biomarkers of IPF [48]. Another limitation of our study was the inability to include early/progressive IPF controls or a validation cohort due to limited tissue availability and the unique end stage pathology exemplified by this cohort. However, we compared the plasma proteome signature from our IPF cohort with a recently published similar signature using the SomaLogics platform with the IPF-PRO registry samples, and identified SPARC, CCL5, CCL17 and CCL22, OLR1 and PDGF-a/B as common biomarkers in both IPF datasets. The IPF-Pro registry cohort had a mean predicted FVC of 69%. In contrast, the mean % FVC of patients in our study was 44%, with a majority of patients at ≤ 30%. It is therefore possible that chemokine and growth factor signaling, immune activation and ECM homeostasis pathways could be consistently dysregulated in early and late stage disease. Notably, our dataset also confirms biomarkers (such as CCL17, PDGF, SPARC) previously identified in early IPF, as well as demonstrates correlation of CXCL12 to lung function, suggesting that many biomarkers identified in our late stage IPF cohort may also be potential early diagnostic markers.

## Conclusions

In summary, we have presented a unique comparative transcriptome-proteome signature of advanced IPF and identified key tissue and circulating biomarkers that could be predictive of progressive/worsening IPF. Further validation of these findings in larger cohorts will help develop a comprehensive panel of biomarkers with clinical utility to address the current unmet need in the diagnosis and management of IPF.

## Supplementary Information

**Additional file 1.** Differential protein expression in plasma of IPF patients compared to healthy controls.

## Data Availability

All data generated or analyzed during this study are included in this published article (and its additional information files). The RNA-seq data is deposited in the GEO database (GSE134692).

## Abbreviations

FC: Fold change
FDR: False discovery rate
IPF: Idiopathic pulmonary fibrosis
ALI: Acute lung injury
ECM: Extracellular matrix
